# Causes of metabolic syndrome and obesity-related co-morbidities Part 1: A composite unifying theory review of human-specific co-adaptations to brain energy consumption

**DOI:** 10.1186/2049-3258-72-30

**Published:** 2014-09-01

**Authors:** Anne-Thea McGill

**Affiliations:** 1 School of Population Health and Human Nutrition Unit, Faculty of Medicine and Health Sciences, University of Auckland, Private Bag 92019, Auckland 1142, New Zealand; 2 B-Med Weight Control Consultancy, Auckland, New Zealand

**Keywords:** Metabolic syndrome, Obesity-related co-morbidities, Theory review, Evolution and nutrition, Food micronutrient, Malnutritive obesity (Malnubesity), Cortico-limbic-striatal, Food addiction, Nuclear factor-erythroid 2-related factor 2 (NRF2), Human brain metabolism, Oxidative stress, Metabolic inflammation

## Abstract

**One line summary:**

Metabolic syndrome and obesity-related co-morbidities are largely explained by co-adaptations to the energy use of the large human brain in the cortico-limbic-striatal and NRF2 systems.

The medical, research and general community is unable to effect significantly decreased rates of central obesity and related type II diabetes mellitus (TIIDM), cardiovascular disease (CVD) and cancer. All conditions seem to be linked by the concept of the metabolic syndrome (MetS), but the underlying causes are not known. MetS markers may have been mistaken for causes, thus many treatments are destined to be suboptimal.

The current paper aims to critique current paradigms, give explanations for their persistence, and to return to first principles in an attempt to determine and clarify likely causes of MetS and obesity related comorbidities. A wide literature has been mined, study concepts analysed and the basics of human evolution and new biochemistry reviewed. A plausible, multifaceted composite unifying theory is formulated.

The basis of the theory is that the proportionately large, energy-demanding human brain may have driven co-adaptive mechanisms to provide, or conserve, energy for the brain. A ‘dual system’ is proposed. 1) The enlarged, complex cortico-limbic-striatal system increases dietary energy by developing strong neural self-reward/motivation pathways for the acquisition of energy dense food, and (2) the nuclear factor-erythroid 2-related factor 2 (NRF2) cellular protection system amplifies antioxidant, antitoxicant and repair activity by employing plant chemicals, becoming highly energy efficient in humans.

The still-evolving, complex human cortico-limbic-striatal system generates strong behavioural drives for energy dense food procurement, including motivating agricultural technologies and social system development. Addiction to such foods, leading to neglect of nutritious but less appetizing ‘common or garden’ food, appears to have occurred. Insufficient consumption of food micronutrients prevents optimal human NRF2 function. Inefficient oxidation of excess energy forces central and non-adipose cells to store excess toxic lipid. Oxidative stress and metabolic inflammation, or metaflammation, allow susceptibility to infectious, degenerative atherosclerotic cardiovascular, autoimmune, neurodegenerative and dysplastic diseases.

Other relevant human-specific co-adaptations are examined, and encompass the unusual ability to store fat, certain vitamin pathways, the generalised but flexible intestine and microbiota, and slow development and longevity.

This theory has significant past and future corollaries, which are explored in a separate article by McGill, A-T, in Archives of Public Health, 72: 31.

## Background

The major health problems that beset almost all populations in the 21^st^ Century are degenerative disorders such as type II diabetes mellitus (TIIDM), atherosclerotic cardiovascular disease (CVD) and cancer. These diseases are strongly associated with certain human behaviours and societal organisation. They also relate to resources available, how and what discoveries have been made and/or technologies invented and employed. In turn, all of these factors depend on the evolution of up-right posture, bipedalism, prehensile forelimbs freed from locomotion and obviously a large, complex brain. Less known is just how much the high energy consumption of such a large brain may have promoted human specific co-adaptations. Unusual characteristic in humans such as slow development, growth and healthy longevity or the ability to gain very large amounts of fat in adipose tissue may be related to brain energy use.

## The metabolic syndrome problem

Over the last few decades rates of the above mentioned diseases, and many other degenerative conditions including liver, kidney, gut, eye, and brain disorders, have increased. This degeneration of organs tends to be associated with central/upper body fat accumulation, hypertension, dyslipidaemia and hyperglycaemia. This well-known risk marker cluster, denoted as the metabolic syndrome (MetS), predicts the development of the above conditions and is epidemic in westernised populations [1].

**Figure 1:**
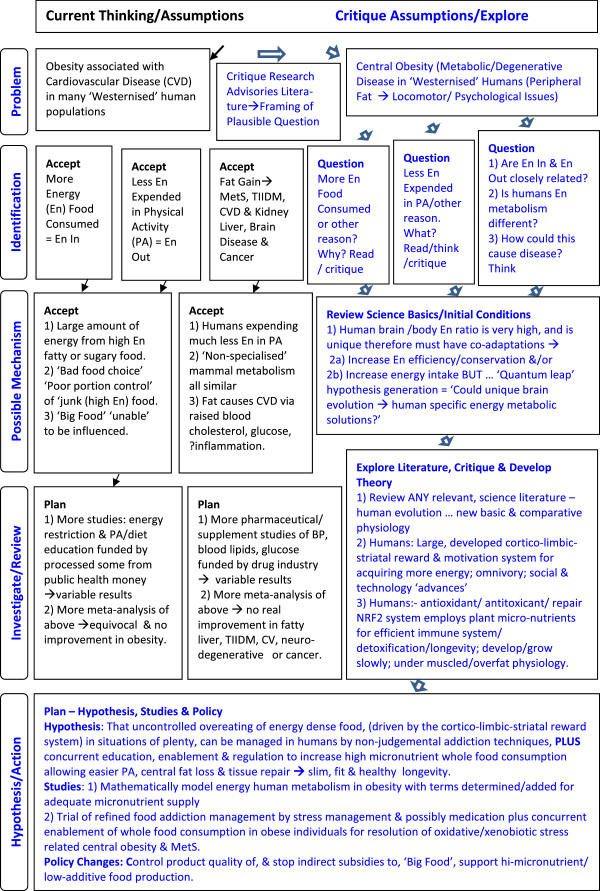
**Comparisons of approaches to solving obesity-related disease.** This figure shows a comparison of methods of enquiry in research and why sometimes meta-analyses are the wrong approach such as when the research question itself needs reconfiguring.

Surprisingly, the basic causes, mechanisms of action and treatment of these diseases are still poorly understood. Addressing ‘inadequate hypotheses and therapeutic mechanisms’ [2] or unsubstantiated assumptions of MetS, may allow linked propositions, derived from various disciplines, to be formulated into a composite unifying theory (Figure 1).

### Assumptions

Briefly, the relevant assumptions held by the scientific and clinical communities, and their funders, have been that: human fat gain occurs simply when ‘energy intake (food) is greater than energy output (physical activity) x metabolic rate’ without fully investigating what contributes to ‘metabolic rate’ [3] (although this may be changing [4]); human (energy) metabolism is typical of similar sized mammals, and unspecialised; obesity occurs in those with poor ‘self-control’, who make ‘bad lifestyle choices’ and need to take ’personal responsibly’ for their condition [5]; intermediary MetS signs and symptoms or markers should be pharmaceutically ‘normalised’ rather than searching for basic causes; and lastly, technological developments in mass food production, pharmaceuticals and medical devices, energy saving devices/transport are good and necessary ‘human progress’ and this justifies resisting inquiry into, or remediation of, any resulting health or environmental harms (Figure 1).With respect to the components of MetS it is assumed that: 1) TIIDM and CVD are mainly issues of excess energy molecule concentrations of glucose and lipids (triglycerides and cholesterol) in the blood. Therefore, treatment should be firstly by specific hypoglycaemic and cholesterol normalising medication, and also by decreasing dietary lipids and sugars, 2) hypertension required similar approaches; specific hypotensive medication, with added dietary salt reduction, and behavioural changes to increase physical activity 3) central obesity, which has increased dramatically, does not need medical therapy as overeating and under-exercising are seen as primarily psycho-sociological behavioural problems (Figure 1).

Weight loss programmes have involved low-energy diet prescriptions, varying with ‘new’ research [6], often-unsuitable exercise programmes, and behavioural modification/motivation therapies. Public health programmes have concentrated on ‘healthy lifestyle messages’ [5] rather than the socioeconomic and commercial regulatory environment, similar to early smoking cessation campaigns. The above approaches are deemed to ‘fail’ due to ‘poor individual compliance’.

Weight loss is metabolically complex. Efficacious, ethical medication development has been erratic and expectations may be unrealistically high compared with those for established hypertension or dyslipidaemia. Furthermore, peripherally obese individuals, usually pre-menopausal women, are significantly protected from metabolic syndrome [7], yet are over represented as private bariatric surgery patients.

With health issues, where studies keep on resulting in equivocal findings, it behoves the research community to spread a review back over the research history and evolution. Widening the enquiry is required to traverse the many fields of the basic science of biological systems and mechanisms. It is very important to sift out areas of political or financial influence [8,9] in the pursuit of scientific or biological plausibility [10] (Figure 1).Any unifying theory that unsettled such deep-seated assumptions on MetS therapies would need start from the basics of human behaviour and physiology. Two major systems, modified in human-specific ways, appear to be contributing to the problem of obesity-related metabolic syndrome, and degenerative disease in general (Figure 1).

## ‘Dual System’ human specific co-adaptations

Human evolution - the physical remains, artefacts, and the environment shaping it - can now be examined using advanced technology. Current biochemical techniques, such as high through-put microarray data analysed using computer assisted mathematical modelling, are applied. This allows studies of nutrigenomics (nutrition-influenced products of genes, proteins) and metabolomics (other chemicals produced from specific cellular processes). This archaeological evidence is then compared with data from the recent era. Thus ‘genomic archaeology’ literature provided the basis, and evidence, for the ‘dual system’ composite unifying theory.

**Figure 2:**
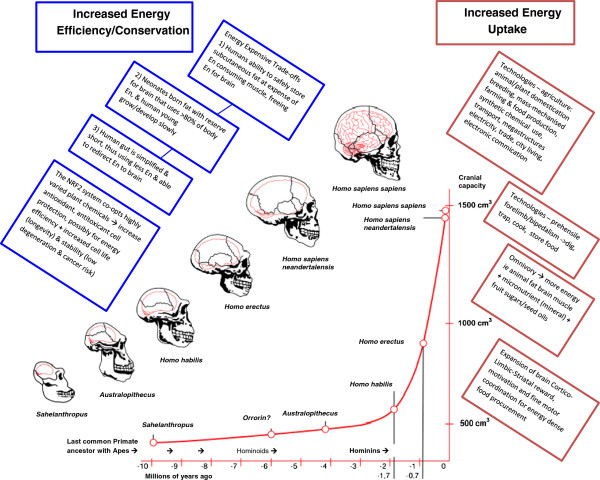
**Human brain enlargement and probable co-adaptations to manage increasing energy use.** Progressive encephalisation meant increased energy consumption by the human brain. Co-adaptations were required, to conserve, and economise, on energy and increase energy uptake. The blue boxes indicate various human-specific adaptations to conserve energy, and the red boxes, to increase energy uptake. Figure adapted from resources at the Muséum d’ Aix-en-Provence 2001 [12].

During human evolution the marked increase in brain size had significant energy use implications [11] (Figure 2). In order to accommodate the brain’s uniquely high energy requirements, it is hypothesised that various human-specific, unusual co-adaptations developed to increase dietary energy and/or conserve body energy use. Two unrelated co-adaptations have co-dependant mechanisms with respect to contributing to MetS and obesity related comorbidities. They comprise a ‘Dual System’. These are the cortico-limbic-striatal and the nuclear factor-erythroid 2-related factor 2 (NRF2) systems (Figure 2).

a) *The Expanded Cortico-Limbic-Striatal System*

The first part of the ‘dual system’ composite unifying theory is that the human cortico- limbic-striatal [13] system expanded during encephalisation. This was largely in order to drive intense efforts into acquiring energy dense food to provide large amounts of glucose for the brain. The cortico-limbic-striatal system involves a "reward" pathway beginning in the old reptilian-mammalian brain ventral tegmental dopaminergic neurons. These connect the ‘motivation-to-act’ limbic system via the nucleus accumbens to the coordinating (and emotional) medial prefrontal cortex [14]. This pathway links the basic needs of a mammal with motivation and (fine) motor control behaviours to satisfy these needs, with frontal lobe time and space coordination [15].

**Figure 3:**
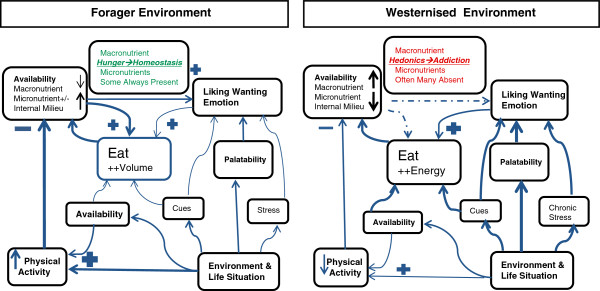
**Influences on eating behaviours and nutrient balance.** In the Forager (hunter/gatherer) environment, humans were often driven by hunger to physically work hard to acquire enough energy and achieve homeostasis, and food in general to maintain their weight and health. Rarely, was refined energy dense, highly palatable food present for long periods or in quantities to elicit addiction patterns via cortico-limbic-striatal system. On the other hand, Westernised environments are characterised by chronic stress, lack of physical activity (green), along with commercial advertising that strongly cues the consumption of easily available, highly palatable, refined food (red). This over-palatable food, is sought for the taste reward or hedonics rather for homeostasis. However, for many people in this environment control of refined, energy dense food is lost and addiction behaviour supervenes (blue). The lack of micronutrients and high, uncontrolled energy input increases fat deposition. Oxidative and general stress, and metabolic syndrome, develop. Figure adapted from Zheng et al. 2009 [16].

The attainment of high-energy food signals the neural reward, over and above the basic homeostatic or maintenance appetite system. A strong, positive memory of pleasure, or hedonia, is laid down for repeating the effort for future food supplies (Figure 3). The food item, and associations with it in time and place, becomes highly memorable; the item assumes a high salience value [16,17].

Intriguingly, there is evidence of recent and continuing genetic variability and evolution, and continuous epigenetic modification of the controlling dopamine transporter [18]. Polymorphisms and mutations in dopamine metabolism can be significant determinants in psychiatric disease.

It is important to recognise that this cortico-limbic-striatal system was likely to have developed to initiate and maintain the drive to pursue lifesaving energy consumption in stressful environments with lack of food. Thus, most physical or psychological stress hormones, neurotransmitter and cytokine pathways are linked to the cortico-limbic-striatal system. The hypothalamic-pituitary-adrenal axis, which results in the secretion of glucocorticoids, and the autonomic nervous or sympathoadrenal medullary and parasympathetic systems [13], are intimately involved.

These stress chemical pathways act to stimulate the release of energy for tissues that acutely need fuel, but return the system to homeostasis as soon as possible. When muscle is in ‘fight or flight’ action, or inflammatory cells activated after injury, food seeking is suppressed, acutely. However, this changes in chronic injury or illness, psychiatric stresses [19], as well as in psychosocial stress, often due to social hierarchical power imbalance [20]. Stress neurotransmitters and pathways link energy metabolism, oxidative stress and MetS to the cortico-limbic-striatal system. In such situations any reward on physically seeking and attaining high energy food becomes ‘corrupted’ into poorly controlled desire for ‘comforting’, highly-palatable energy dense food, direct chemical stimulators (drugs of addiction) or behaviours that ‘promise reward’ (gaming and gambling).

Fast though the human cortico-limbic-striatal system has evolved [18], it has not been able to adapt and/or down regulate in the face of exposure to the current chronically stressful, sedentary environments of increasingly refined energy dense food, and ‘reward stimulation’ (Figure 3). Addiction has arisen, and is characterised by obsession and compulsion to seek the item of ‘reward’ or perform behaviours that ‘comfort’ or temporarily calm feelings of anxiety. Unfortunately, these behaviours persist in spite of difficulty and/or damage to health, and social and work functioning [16].

This process sets the scene for addiction behaviours in many people in times of easy availability of over-palatable, energy dense food. This pathway can be directly stimulated by priming neurotransmitter molecules (opiates, amphetamines, cocaine, nicotine cannabinoids and others) that are part of appetite or related pathways. Cues are very important in addiction [21]. Ultimately extremely hard-to-break habits form, where there is often no pleasure, but a reward hypo-function or deficiency syndrome develops [22,23]. Withdrawal symptoms maintain the repetitive behaviour [16].

Interestingly, with respect to treatment, the dopamine based cortico-limbic-striatal system does not ‘link with logic’, and perhaps actively resists analytical thinking [14]. In contrast, the serotonin pathway is widespread in the cortex and can be affected by cognition and logical thinking [14]. Hence reasoning with people addicted to items or behaviours has a low rate of success.

Long term assistance in low stress (non-judgemental) therapeutic environments needs to be provided. For example, support with pre-planning rules around abstinence of addictive foods/drugs/alcohol/, and encouragement to replace such items by moderately appetising high nutrient food/less problematic drugs/behaviours can result in modest remission from addiction. As expected, depending on circumstance, there is often a fluctuating course or repeated cycles, and variable levels of success [24], but hope for future improvements as understanding of food addiction grows [25].

Refined energy food was possibly the first addictive ‘dangerous consumption’ [26]. The concept of addiction in general, and refined energy food addiction specifically, has been validated by much recent experimental and clinical work [13,27,28].

Additionally, the cortico-limbic-striatal system has been behind the drive to develop technology and social systems to grow, breed, process, refine, store, transport, trade, market and consume highly palatable, energy foods or macronutrients (carbohydrate, fat, protein and alcohol). At the same time industrial power brokers, possibly ‘power addicts’ themselves [20], are fostered, and favour systems that exploit ‘addicts’. Unfortunately, poor countries that are exploited for cheap resources and/or labour often have compounding high rates of corruption and wealth disparity [29]. Regulation of processed food, tobacco, alcohol and other drugs of addiction can be minimal [30].

Addiction leads to neglect of normal, healthy behaviours; the consumption of adequate low processed plant food is deprioritised, and fails. Humans develop seemingly-imperceptible insufficiency of various nutrients. These are the micronutrients, composed of minerals, vitamins and many classes of useful plant biochemicals, or phytonutrients, the latter of which are still being characterised. Whilst it had been thought that pure overload of sugars and fats/oils are responsible for obesity, the omission of large varieties and volumes of dietary micronutrients may also contribute, and leads to next part of the theory.

Conservation of body energy is hypothesised to occur via increasing the efficiency of oxidant buffering or antioxidant effects during macronutrient oxidation. In humans, possibly related to high energy flux in the brain [31], antioxidants are very active in unusual ways. Both urate (the predominant serum antioxidant, and raised in humans) and vitamin C (obligatory in the human diet) metabolism is unusual in humans, possibly having profound roles in foraging behaviour, in the face of starvation and thirst [32].

b) *The Micronutrient-Dependant Nuclear Factor-Erythroid 2-Related Factor 2 (NRF2) System*

**Figure 4:**
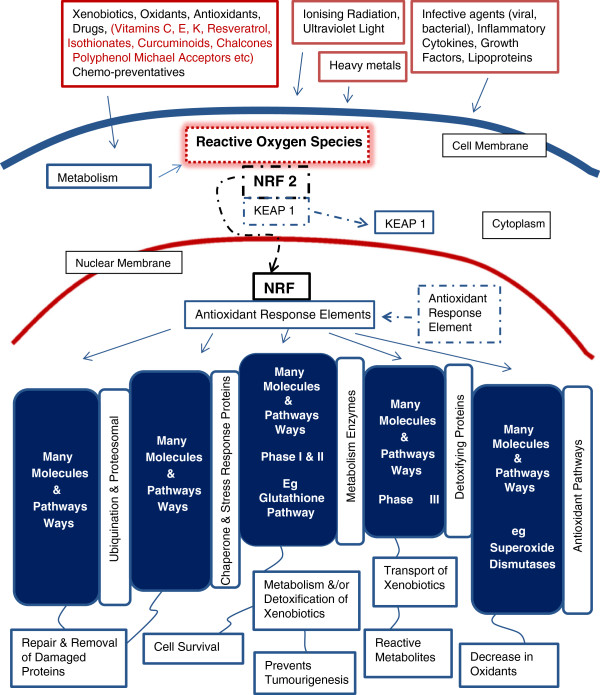
**Normally functioning human NRF2 system.** The nuclear factor-erythroid 2-related factor 2 (NRF2) dissociates from the Kelch-like ECH-associated protein 1 (KEAP1) and migrates to the nucleus on a wide variety of variably noxious, often pro-oxidant stimuli. The appropriate responses are then elicited and magnified via pathways sets for antioxidant, antitoxicant and repair cell protection. This principle of slightly negative stimuli causing a restorative response is denoted hormesis. In humans unknown numbers and types of micronutrients are involved in moderating and modulating the NRF2 system. Figure adapted from Jaiswal 2010 [36].

In the last decade or so, cancer research has shown that many antioxidants act through the second part of the ‘dual system’; the NRF2. The NRF2 is the main transcription activator for a cellular system comprising an amplifying cascade of antioxidants/antitoxicants and cell repair pathways or a comprehensive cytoprotection system [33,34] (Figure 4). The NRF2 is present in most cells, and there are specific NRFs in the liver and placenta. The human NRF2 pathways have co-opted, and importantly, come to depend on, a wide selection of phytonutrients as slightly pro-oxidant inducers (for example, vitamins C, E & K), inhibitors, activators, Michael acceptors and stand-alone oxidation buffers [35] (Figure 4). Michael acceptors are molecules that modulate energy reactions by non-specifically, temporarily accepting electrons and returning them on completion of each reaction. They are often complex phytonutrients [35].

The major unknown aspect of this second part of the composite unifying theory is, in fact, a hypothesis in that there is no definite evidence yet, and further studies are required. The unproven hypothesis is that the NRF2 antioxidant and antitoxicant functions associated with high food-micronutrient diets imbue cells with enhanced overall energy efficiency. It is hypothesised in the current paper that the primary reason for the human NRF2’s co-option of large classes of phytonutrients was to develop a new form of energy efficiency in order to free energy for encephalisation. Some modelling studies suggest such energy efficiency in the high-functioning human NRF2 [37]. Vomhof-DeKreya and Picklo Sr state ‘The pharmacological activation of the NRF2 pathway opens the possibility that other dietary NRF2 activators such as the cabbage (brassica) family metabolite, sulforaphane may also have effects on cellular lipid metabolism and total energy expenditure’ [38] (Figure 4).

Notably, in severe inflammation and advanced cancer, cachexia may result from the marked energy inefficiencies associated with high oxidative stress levels, although both conditions coexist with states of lethargy and anorexia. Energy dilution of high fibre fruit and vegetable diets is the usual reason cited for their contributing to normal weight [39].

As humans have become more nomadic and migratory, they have been adapting to countless species of plant foods from many genera, many of which contain well known antioxidants and toxicants, as well as vast numbers of variably chemically-reactive secondary plant molecules. Many of these phytonutrients are members of large plant families containing numerous variants, as seen, for instance, with the sun protection caroteno-retinoids. These phytonutrient split into the carotene and retinoid vitamins, beta-carotene and vitamin A, and carotenals [40], all with different functions.An extremely important aspect of the human experience with this vast array of plant chemicals is the likelihood that at least some or other of the phytochemical variants will be suitable to activate almost all of the human polymorphisms in the wider NRF2 system. A high fruit, vegetable and protein diet, for humans will likely suffice for protecting almost all human cells, without resorting to specific gene-nutrient tailoring (Figure 4).

Continuous high level antioxidant and antitoxicant processes likely provide first tier cytoprotection. The second tier protection is a process of cell organelle recycling (autophagy) to repair long-lived cells that become damaged [41]. Resveratrol, a highly studied, polyphenol phytoalexin, is an antioxidant but also contributes to autophagy [41,42]. The NRF2 system is known to be involved in the liver cell autophagy; liver cells live a medium time, are very metabolically active and also have a liver specific NRF system [43]. It is hypothesised that these processes reduce the need for apoptotic cell death [41]. Thus, irreplaceable long lived cardiomyocytes and neurons, for example, can live and function for a potential 100 year-plus human life span, instead of around the 40 years predicted from mammalian physiology [44].

Concurrently, the exposure to such a variety of foods, especially secondary plant chemicals meant that human metabolism had to manage many foreign, unnecessary or poisonous chemicals (xenobiotics). The NRF2 system developed a complex detoxification system for these xenobiotics that was intimately linked to antioxidants in the diet. Competent, faithful cell replication in fast turnover or short lived cells was enhanced. The NRF2 system is therefore, probably, also at the heart of general immune competence (Figure 4). The human NRF2 has become experienced at processing, and detoxifying where necessary, myriads of chemicals in the natural environment, many of which are antigenic parts of infective agents. A high-functioning cytoprotection system became very important [45,46].

Leukocytes turnover rapidly, in the face of infection or other immune stimulation, and must replicate in a highly controlled, accurate manner, to produce a whole series of situation-specific cytokines and immunoglobulins, without xenobiotic interference. Sulphoraphane for example, confers immune competence for protection against infection/infestation damage, preventing dysregulation and dysplasia of the intestinal and glandular epithelium [47].

Exposure to typical organisms and antigen signals via the high functioning NRF2 pathways prepares T-regulatory lymphoid cells ‘to polarise’ [48] the T helper cells 1 & 2 appropriately. This process results in self and non-self tissue being well recognised. In contrast, the (hyper) hygiene theory [49] hypothesises that, from pre-conception onwards [50], human tissues are not exposed enough, or at a typical developmental period, to natural antigens. Exposures occur with the 1) gut to food, 2) respiratory tract to airborne particles, and 3) skin to environmental ‘contaminants’. Concomitant high use of antibiotic personal ‘hygiene’ products, exposure to home and work place cleaning agents and other industrial chemicals, and food additives, together with chronic low micronutrient intake, probably contributes to suboptimal NRF2 function. Disturbed NRF2 processing has likely lead to increased rates of over-reaction to mild environmental stimuli or allergy. Autoimmune disease is probably faulty recognition of ‘own tissue’, and attempts at its destruction result [45,46,48].

With respect to the central nervous system, there is much evidence that ‘metabolic syndrome or diabetes of the brain’ [51] exists. Inadequate nutrition [52], various toxins [53] and oxidative stress [52] affects glial cells [54] and neurons, causing degeneration [52]. The development of misfolded proteins, neurofibrillary tangles, mitochondrial dysfunction [41] and cell death as seen Alzheimer’s, Parkinson’s and other generalised and site-specific neurodegenerative diseases is well documented.[52] Various micronutrients, especially phytonutrients [55], are shown to ameliorate brain degeneration [52].

It is important to note that the NRF2 system is usually stimulated by slightly noxious, often pro-oxidant molecules or physical damage The system, then reacts and amplifies appropriate cell protection – a process called hormesis [56]. Many vitamins and phytonutrients have a hormetic effect [56], such as resveratrol which, synergistically with other phytochemicals initiates an antioxidant response via typical, slightly pro-oxidant stimulation [57]. However, high dose mono or multivitamins, nutraceuticals, supplements and minerals are likely to flood the NRF2 cell protection pathways and be deleterious [58]. In addition, many supplements do not contain what their labels say, and other anti-nutrient additives are common [59].

Lastly, typical hormetic stimuli such as prolonged, reasonably strenuous exercise with muscle micro-damage [60] and arterial shear stress [61], or inflammation from infection and injury, occur at the same time as energy stores – fat – must be mobilised. These lipids need oxidising in a controlled manner, but often rapidly and in large amounts. Pathway activation involves energy sensing [56], organised lipid release, and energy uptake into, and deployment in appropriate, tissues [38]. Thus energy use, dealing with toxins, inflammation and repair are often concurrent processes that require NRF2 control of the many interlinked pathways (Figure 4).

At this juncture it is appropriate to define the whole food diet, as a diet which could furnish the human NRF2 with adequate ratios of micronutrients to macronutrients. A whole food diet could be defined as a varied, moderate to high fibre volume, micronutrient dense *ad libitum* diet, which includes foods which humans have been eating for most of their evolution: wild and low-input farmed, fresh, cooked, fermented, preserved (heritage where possible) pigmented fruit and vegetables, (oil based) nuts/seeds and high protein/fibre seeds (pulses), mixed free-range animal products (muscle, fat and offal). A whole food diet excludes addictive, energy dense foods (except for rare occasions): significantly processed, refined foods, especially highly bred, energy dense cereal grain/cane and tubers/beets and their starch and sugar derived products, and industrially modified plant oils, with preservatives and other ‘chemical additives’.

Note that so called carbohydrate ‘staples’ only date from agricultural times. Highly bred, ‘energy crops’ that yield ‘pale coloured’ foods such as cereal (wheat, barley, rice maize) flour products (leavened and unleavened breads, pasta, biscuits/crackers) are such products. They are often combined with table sugar/syrups and/or salt [23] and refined fat or oil (cakes, pastries and confectionary). They all tend to be addictive; their consumption hard to control. Adequate energy for most individuals can be consumed from, and can largely be replaced by, minimally processed higher protein foods, fats/oils, and higher fibre carbohydrates naturally found in foods not grown solely for energy.

In summary, unique human physiologies involved in energy-expensive encephalisation involve equally unusual energy acquisition and conservation co-adaptations, which appear to, result in post-technological ‘nutritional and metabolic clashes’. Furthermore, there are other human-specific adaptations that contribute to the composite unifying theory on MetS.

### Other human-specific metabolic Co-adaptations - part of the composite unifying theory

There are other unusual metabolic human co-adaptations, apart from the two discussed above, which bear on the development of obesity, and which are rare in mammals.

Firstly, humans exhibit extremely slow growth and development for mammals of their size, possibly to spare energy for the glucose-hungry brain [62]. The extended human lifespan is associated with delayed puberty, and the unusual occurrence of menopause and healthy life for many years after breeding, in females, ceases [63]. This slow development and long life is likely to be largely dependent on the hyper-efficient cell protection mechanisms of the NRF2 systems exposed to a high micronutrient diet. These mechanisms comprise augmented antioxidation and repair afforded to long lived cells, as well as replication stability provided by antitoxicant action, especially in glandular epithelium and leukocytes.

Humans have unusual antioxidant pathways and levels that interact with macronutrient metabolism. Hominoids lost the enzyme of the last step in vitamin C synthesis in the pre-Miocene(18–23 million years ago) era, thus vitamin C is required in human diets. The same ape stock lost a functional uricase to metabolise uric acid to allantoin, so uric acid levels are higher than most other mammals [32]. Fructose depletes adenosine triphosphate (ATP), thus uric acid formed from the adenosine. Concurrently, triglycerides are also formed from fructose via the uncontrolled hexokinase-catalysed reaction [64]. The liver and central organs rapidly fill with fat, although the reaction is inhibited by vitamin C. This may be an energy conservation strategy as hominoids gorged on ripe, temperate, fructose replete (but vitamin C deficient) fruit. High levels of uric acid may have been produced in autumn in order to survive the cooler, drier winters in the peri-Miocene period [32]. Increased insulin resistance and energy uptake can also be promoted by raised levels of the uric acid. Uric acid is also synthesised from purines on muscle catabolism in starvation, and stimulates foraging once fat stores are depleted. Transfats and alcohol have a similar metabolism in that they are metabolised in the liver, not regulated by insulin, and do not form glycogen [4].

Note that high levels of glucose have been shown to produce fructose via the polyol pathway. When large amounts of sucrose are metabolised to glucose and fructose rapid, large quantities of fat are produced and overload liver cells [64]. This process is extreme in populations that drink high levels of fructose from high fructose corn syrup. In addition, it is now understand that liver fat increases, in those who consume excess sucrose in any form, and, by extrapolation, who consume large amounts of starch [65]. Interestingly, most fruit is not eaten when overripe, so vitamin C and unknown numbers of phytonutrients are present. There is evidence that fructose and glucose do not pose a problem when eaten in natural fruit with the skins and micronutrients present [32]. This is likely to be especially true for old fashioned, heritage plant foods, with no artificial fertiliser or pesticides applied, as they continue to synthesise plenty of defence secondary chemicals to deal with microbial, infections. Thus organic ripe grapes infected by yeasts produce resveratrol and many other phytoalexins and useful nutrients, during the wine fermentation process.

Another co-adaptation is the various forms of energy-expensive-tissue trade-off that may have developed in humans. Such trade-offs involve one organ or system decreasing in size, complexity and/or function to allow energy to be directed instead to another organ, in this case the brain.

The relatively short, simple human intestine has been proposed to be an example, although the human gut to brain trade-off is disputed [66]. However, the human omnivorous gut is very adaptable and flexible. The exposure to vast numbers of phytochemicals requires that the lower intestine has a great diversity in microbial biotype. An increased *Bacteriodetes-*dominant microbiota associates with healthy human populations. Such human groups consume a high plant, and low processed and energy food, diet and are exposed to less industrial toxins and antibiotic medication [49,67,68]. In obesity and TIIDM, excess energy harvest is postulated to occur in the less healthy *Firmicutes*-dominant colon. This may be pathological as food energy intake is typically high in these conditions. Addtionally, significant quantities of inflammatory microbial debris and DNA are found in the plasma. Some beneficial bacteria such as *Lactobacillus* tend increase energy uptake in the slim, but decrease it in obesity [68]. Further, healthier diets and weight loss in obese individuals return the gut microbiota towards a healthier diversity [68]. In the western diet there is chronic energy overload with large amounts of excess fructose, and oligo- to poly-saccharides present in plants highly bred for energy and sweetness. This dietary pattern has been associated with excess gas distention and may contribute to the irritable bowel syndrome [69]. However the lack of non-fermentable fibre and micronutrients in this dietary pattern, added fermentable oligosaccharides in ‘diet products’ (including sorbitol in chewing gum and toothpaste) and ingested low grade man-made toxins probably also contribute to the *Firmicutes* dominant biotype and irritable bowel. Whilst treating symptomatic intestinal infections may be valid, it may be best to not encourage antibiotics in underfed children to promote growth, especially if weight is prioritised over height [70].

Another expensive energy trade-off is high metabolic rate-muscle being traded for lower metabolic rate fat or adipose tissue [71]. This is an interesting three way trade of muscle bulk for high energy content adipose which in turn provides an energy store or buffer for the brain. Lipid accumulation in metabolically-safe subcutaneous hip/buttock/thigh adipose tissue depots [72], can be very large in some human individuals or groups [73]. Such adipose tissue stores may be especially important for mobilisation during pregnancy and lactation [74], where there are two brains to supply. Furthermore, humans are born relatively fat. As only one of two species which delivers fat infants [75], human neonates have brains which consume an extreme >85% of the body’s energy [11].

### Malnubesity

The current cohort of humans has been unable to efficiently oxidase their excess energy. This leads to ectopic, toxic lipid accumulation [76] in perivisceral, upper body and organ tissue, as seen with central obesity. This state of excess oxidative stress and ineffectively detoxified xenobiotics [77] in the suboptimal, micronutrient-deprived NRF2 system leads to the inability of cells to perform maintenance and autophagic repair work [41]. Fast-turnover cell replication loses its strict regulation and tends to dysplasia, with immune cells particularly being prone to acquired DNA damage; immune dysfunction and thence higher rates of infection, and malignancy [78].

Metabolic inflammation (metaflammation [79]) occurs, especially in the arterial endothelium (as atheroma containing oxidised, pro-thrombotic, lipid engulfed by cytokine-secreting foam-cells), endocrine epithelium (dysplasia), liver (production/secretion of inflammatory proteins and dysplasia), cardiomyocytes (ischaemia/lipotoxicity [76])) and in the central nervous system damaged and misfolded or glycated proteins are deposited (neurodegeneration [41,80]). Many other related mechanisms occur, often overlapping in different tissues [4]. This could be called malnutritive obesity or malnubesity [81].

The rapid co-development of the large, powerful brain, bipedalism and prehensile upper limbs, and germane to this thesis, requisite nutritional and energetic co-adaptations, produced an increasingly versatile organism. *Homo species* became a successful nomadic, social forager*,* becoming *H. sapiens sapiens* or modern human about 200,000-75,000 years ago [82].

## Summary

In summary, it is proposed that as the human brain became enlarged, which increased its energy demands on the body, a human specific ‘dual system’ and other co-adaptations were required to provide extra energy for the brain.

To increase energy uptake, the cortico-limbic-striatal system, an expanded neural network, has driven humans to devise wide-ranging technologies to make available extremely refined, high energy food for the brain. The same system is probably involved in addiction, initially to refined energy food, and it is also is responsible for all addictions when the items are highly available. Note that it is the same area of the brain area of the brain responsible for compulsions to seek, to the extent of perseveration through great hardship, rewards of great peer acclaim or self-satisfaction. The cortico-limbic-striatal system drives competition in enterprises such as climbing the highest mountain, building the tallest building, designing the most widely marketed drug, but probably also applies to ‘addiction to power’ over resources and other humans [20].

Ultimately, technologies have processed food so it is unrecognisable, with large quantities of micronutrients eliminated and many untested, anti-nutritive chemical additives, leaving refined energy dense, unhealthy foods. However, humans depend on micronutrient dense food for their gut microbial, and therefore their own, health and longevity. The NRF2 system’s power to maintain an extremely high level of antioxidant cell protection and detoxify persistent man-made chemicals depends on relative and absolute food micronutrient sufficiency.

## Conclusion

The composite unifying theory includes the ‘dual system’ theory and other human specific co-adaptations, as an explanation for malnubesity, a condition of excess fat accumulation which however has a concomitant insufficiency of vitamins, minerals, plant and other micronutrients. An understanding of the composite unifying theory can be used as a basis to remediate the current MetS epidemic.

The second article of the two in this issue of Archives of Public Health, on the composite unifying theory on causes of MetS and obesity-related morbidities [83], reviews the corollaries of the various parts of the theory. In order to ‘test’ this composite unifying theory it is important to show that the hypothesis and sub-theories pertain throughout the whole of human evolution and history up till the current era, and the overall theory is generally congruent with high quality research data.

Firstly, current and past deficient micronutrient and/or malnutrition (starvation) scenarios are re-interpreted. Secondly, the effects of man-made pollutants on degenerative change are examined. Lastly, projections are made from current to future patterns on the state of ‘insufficient micronutrient and/or unbalanced high energy malnutrition with concomitant ectopic lipid deposition, central obesity and metabolic dysregulation’ or ‘malnubesity’.

Forecasts on human health are made on positive, proactive strategies using the unifying theory, and compared with the outlook for humans on maintaining current assumptions and the status quo. Areas of further research are outlined. A table of suggestions for possible public health action is included.

### Glossary of Terms

Hormesis – a system dependant on contact with small amounts of variably noxious stimuli which then reacts, repairs damage, with mitohormesis being cell replication or growth on such stimuli, and ‘adaptive repair’ meaning that continuous stimuli results in more permanent defensive change so as to be better protected from the stimulus in future.

Malnubesity - a state of insufficient micronutrient and/or unbalanced high energy, malnutrition, causing ectopic lipid deposition in organs, central obesity, and metabolic dysregulation/syndrome.

Metabolic syndrome – a set of markers originally derived from the classic ‘cardiovascular risk factors’ consisting of hypertension dyslipidaemia and hyperglycaemia or type II diabetes mellitus, with central obesity and other disease indicators now included, an also used for risk prediction of oxidative stress and low grade inflammation related chronic conditions such as kidney, liver, neurodegenerative disease and cancer.

Metaflammation – chronic low grade inflammation, which may be stimulated by oxidative stress, associated with metabolic disturbance, and without an apparent productive immune role.

Nutrition transition – the change from mainly healthy traditional foods and their production and preparation methods, to the commercially processed, micronutrient depleted, energy dense foods derived from Western European cuisine, and which is associated with excess weight and metabolic syndrome.

Obesogenic – causing obesity as in ‘obesogenic environment’ a physical social, psychological and nutritional situation that makes unhealthy fat gain a likely outcome.

Westernised – highly technological consumerist, hedonic and commercial patterns of life, originally derived from western Europe and Britain, but adopted by or foist upon populations which usually retain variable levels of their original political and cultural systems, although often highly blended with new technologies.

Phytonutrients and Phytoalexins – phyto or plant, nutrients are ‘useful’ chemicals that appear to confer health in humans and belong to many large groups of complex chemical families, such as terpenoids (carotinoids), phenols (flavonoids eg tea catechins, soy genistein polyphenolic phytoestrogens eg wine resveratrol flaxseed lignans), alkaloids (eg caffeine) organosulphurs and others. Phytoalexins are plant secondary pathogenesis or defence chemicals that are only synthesised at certain times for specific purposes such as, to prevent plants succumbing to water stress (osmotins), for defence against microbes, anti-feedants/contraceptives for grazing animals, and are usually quite reactive in mammalian metabolism.

Xenobiotic - foreign poisonous or unnecessary chemicals (some are formed in the body) that damage cells or render them dysfunctional.

## Abbreviations

CVD: Atherosclerotic cardiovascular disease; MetS: Metabolic syndrome; NRF2: Nuclear factor-erythroid 2-related factor 2; TIIDM: Type two diabetes mellitus.

## Competing interests

The authors declare that they have no competing interests.
